# Unexpected Effects of Cerebrospinal Fluid on the Prevention of Cerebral Thromboembolism and Blood–Brain Barrier Disruption: First Experimental Study

**DOI:** 10.5152/eurasianjmed.2023.22317

**Published:** 2023-02-01

**Authors:** Mete Zeynal

**Affiliations:** 1 Department of Neurosurgery, Atatürk University Faculty of Medicine, Erzurum, Turkey

**Keywords:** Subarachnoid hemorrhage, choroid plexus, cell death, thromboembolism

## Abstract

**Objective:**

We investigated the presence of thromboembolism that may develop in hippocampal arteries due to decreased cerebrospinal fluid volume because of choroid plexus damage caused by subarachnoid hemorrhage.

**Materials and Methods:**

Twenty-four rabbits were included as test subjects in this study. The study group comprised 14 test subjects administered autologous blood (0.5 mL). Coronary sections of the temporal uncus were prepared to observe the choroid plexus and the hippocampus together. Cellular shrinkage, darkening, halo formation, and ciliary element loss were considered criteria for degeneration. Blood–brain barriers were also examined in the hippocampus. The density of degenerated epithelial cells in the choroid plexus (n/mm^3^) and thromboembolisms in the hippocampal arteries (n/cm^2^) were compared statistically.

**Results:**

Histopathological examination revealed that the number of degenerated epithelial cells in the choroid plexus and the number of thromboembolisms in the hippocampal arteries were 7 ± 2 and 1 ± 1 in group 1, 16 ± 4 and 3 ± 1 in group 2, and 64 ± 9 and 6 ± 2 in group 3, respectively. The significance levels were *P* < .005 for group 1 vs. group 2, *P* < .0005 for group 2 vs. group 3, and *P* < .00001 for group 1 vs. group 3.

**Conclusion:**

This study demonstrates that decreased cerebrospinal fluid volume induced by choroid plexus degeneration causes cerebral thromboembolism following subarachnoid hemorrhage, which has not been previously described.

**Main Points**

- Cerebrospinal fluid (CSF) existence has a critical role in the maintenance blood flow in cerebral arteries.
- A decrease in CSF may result in thromboembolism in cerebral vessels.
- In our study, we have demonstratedthat thromboembolism in cerebral vessels is a result ofsubarachnoid hemorrhage affecting the CSF.

## Introduction

The choroid plexus (CPX) secretes a significant amount of cerebrospinal fluid (CSF), which plays a crucial role in blood–lymph circulation, excretion, nutrition, and the maintenance of endocrine, hormonal, and immunological functions of the brain. In this respect, CPX works in the lungs, liver, spleen, thyroid gland, pancreas, intestines, and kidneys. The volume and content of the CSF must be within the normal range for the healthy maintenance of cerebral blood flow. Decreased blood flow to the CPX leads to decreased CSF secretion.^1^ Cerebral arteries pulse easily in CSF-rich subarachnoid spaces,^2^ and CSF is of great importance for the regulation of osmotic and oncotic pressure. Subarachnoid hemorrhage (SAH) disrupts vascular dynamism and rheological functions, and fat embolism causes poor prognosis in patients with SAH.^3^ If CPX innervation is disrupted by ischemia of the lower cranial nerves, the CSF cannot be secreted, vascular hemodynamics may be disrupted, and fat embolism may develop.^2,4^ Cerebral venous thrombosis is a serious complication of spinal anesthesia. Deep cerebral venous thrombosis may occur in cases of decreased CSF volume due to rhinorrhea, transsphenoidal surgery, spinal surgery, skull base defects, lumbar puncture, or injury of the CPX following central nervous system infections such as coronavirus disease 2019;^5-11^ this is because decreased CSF volume causes degeneration of the CPX.^12^ Cistern irrigation may be beneficial because of the protective effects of cerebral circulation in the treatment of meningitis.^13,14^ Cerebrospinal fluid exchange or cerebral ventricular dialysis can be used as new treatment modalities in the future. In this study, we investigated the hypothesis that CPX desquamation following SAH may cause decreased CSF pressure and consequently thromboembolism in hippocampal arteries.

## Materials and Methods

Experimental research ethics committee approval (no. E- 42190979-050.01.04-220036049) from Atatürk University Faculty of Medicine, was obtained. The experiment was performed with 24 male New Zealand white rabbits, which were divided into a control group (n = 5, group 1), sham group (n = 5, group 2), and study group (n = 14, group 3). Inhaler isoflurane was administered to test subjects, and subcutaneous ketamine HCl (150 mg/1.5 mL) and xylazine HCl (30 mg/1.5 mL) combination was administered for general anesthesia. Blood from the auricular artery (0.5 mL) was obtained and injected into cisterna magna of group 3 test subjects in 1.5 minutes by using a 22-gauge needle for making experimental SAH. In group 2, physiological serum (0.5 mL) was injected into the cisterna magna.

After euthanasia, brains were removed and stored in 10% formalin solution for 7 days. Samples were embedded in paraffin blocks in consecutive sections to evaluate the number of thromboembolisms in the hippocampal arteries of the middle cerebral arteries and the density of degenerated epithelial cells in CPX. Twenty consecutive sections were prepared from each of the 5 horizontal brain sections (1 mm) at the level of the middle cerebral arteries. Hematoxylin, eosin, and Glial Fibrillary Acidic Protein (GFAP) stains were used for the CPX and hippocampal tissues. The specimens were examined under light microscobe, and stereological methods were used to determine the epithelial cell densities of CPX. Histopathologically, cytoplasmic condensation, nuclear shrinkage, cellular angulations, and pericytoplasmic halo formation secondary to cytoplasmic regression were considered cellular degeneration criteria. The blood–brain barrier formed by the hippocampal arteries and surrounding astrocytes was partially impaired in group 2 and severely impaired in group 3. While partial endothelial damage and moderate fragmentation of astrocytic extensions were observed in group 2, significant endothelial damage and a high degree of fragmentation of astrocytic extensions were observed in group 3.

### Statistical Analysis

The number of occluded branches and degenerated CPX epithelial cells were statistically compared. One-way analysis of variance followed by Bonferroni’s post-hoc test was used to determine significant differences between groups. Differences were considered statistically significant at *P* < .05.

## Results

### Clinical Results

Two test subjects of group 3 died between days 5 and 8. Loss of consciousness, nuchal rigidity, convulsions, apnea, cardiac arrhythmia, and respiratory disorders were observed in the test subjects that died later. Intraventricular blood leakage was detected in 2 dead test subjects and in 3 surviving animals. Cardiorespiratory rhythm disturbances, fever, nuchal rigidity, confusion, pupillary diameter changes, and urinary and fecal retentions were observed. Two animals died due to cardiorespiratory arrest.

### Histological Findings of the Choroid Plexus

Choroid plexus contained epithelial cells lined with brush-edged cuboidal epithelium surrounded by thin stroma with numerous villi and large capillaries. Epithelial cells of the CPX had round, centrally located nuclei in animals of the control group. There were clear signs of nuclear condensation and cell body shrinkage in group 3, suggesting apoptosis of the epithelial cells. The mean cell density of CPX was measured using stereological methods and found to be 48.236 ± 6432/mm^3^ in group 1. Normal epithelial cells were 12.95 ± 3.14 µm in height and apical microvilli were 3.10 ± 0.71 µm in height. Apoptotic cells were detected using GFAP staining. Dark GFAP-positive cells in the CPX group were abundant in group 3. Choroidal artery spasms, cell injury in choroidal ependyma, apoptosis in choroidal cells, thickening of pia-arachnoid, meningeal adhesions, and blood cell density in the subarachnoid spaces were more severe in test subjects with CPX degeneration.

**Figure 1.**
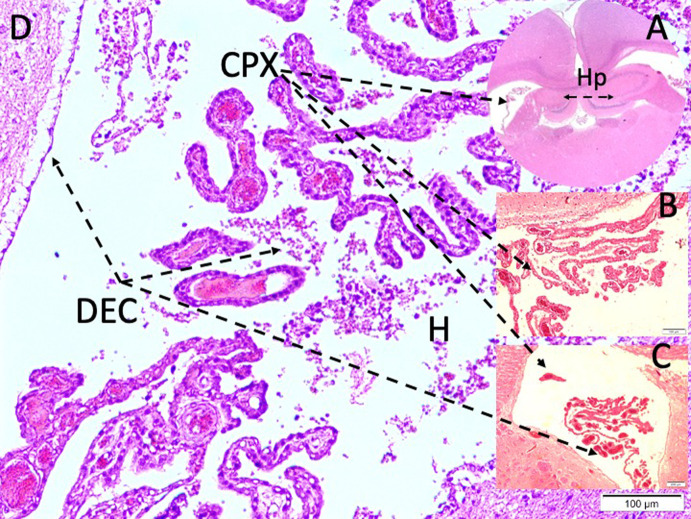
In the control group, the choroid plexus (CPX) and hippocampus localization in the lateral ventricle is observed. Mild degeneration in the CPX is observed in the sham group in a ventricle filled with degenerated ependymal cells, and blood cells are more atrophic and have lost their ciliary appendages in the study group.

**Figure 2.**
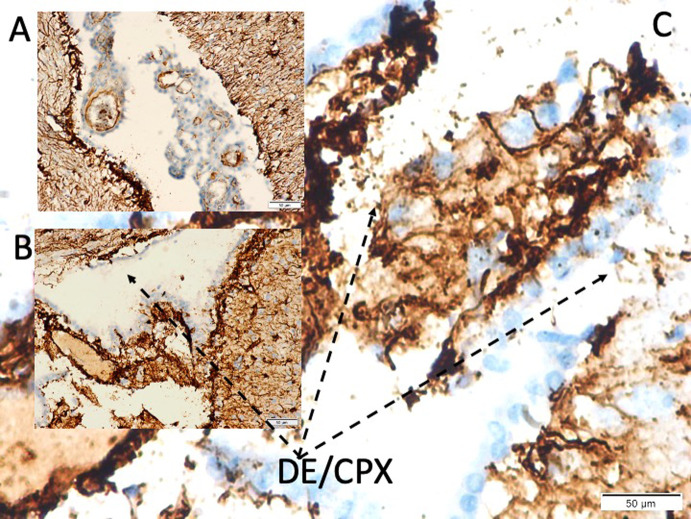
In the control group, normal choroid plexus (CPX) is observed in the lateral ventricle, and mild degeneration is observed in the sham group. In the study group, atrophic CPX, damaged/missing ciliary appendages, degenerated ependymal cells with dark nuclei, and dense cytoplasm are observed in a dilated ventricle.

**Figure 3.**
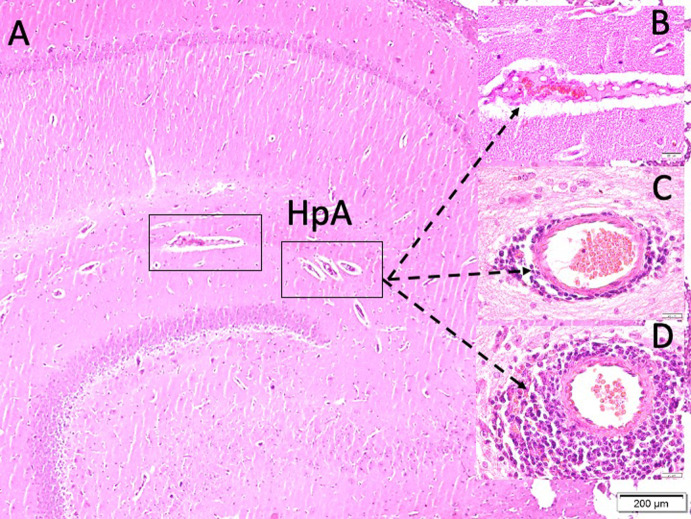
In the control group, the hippocampus is observed together with its neuronal layers and arteries, and a normal artery in the perivascular space is filled with clear cerebrospinal fluid. A slightly narrowed artery surrounded by a few defense cells is seen in the sham group, and a significantly narrowed artery surrounded by many defense cells is seen in the study group.

**Figure 4.**
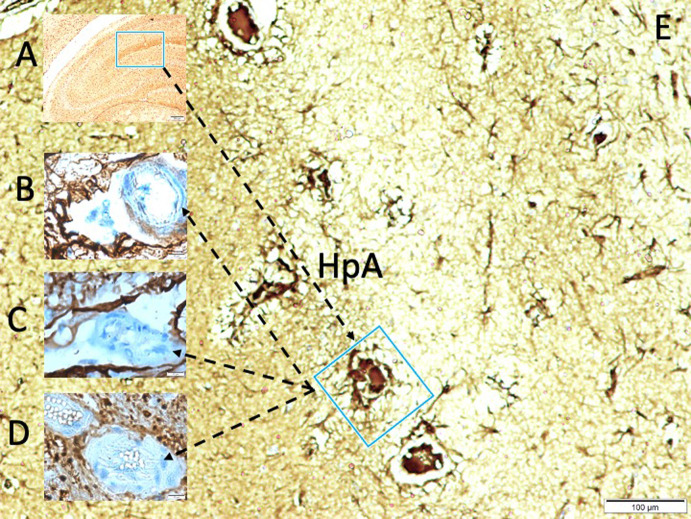
The appearance of the hippocampus and arteries is normal in the control group. A normal hippocampal artery in perivascular space filled with transparent CSF is seen in the control group. A slightly narrowed artery surrounded by few defense cells is seen in the sham group, and a significantly narrowed artery surrounded by a large number of defense cells is observed in a significantly narrowed perivascular space in the study group.

**Figure 5.**
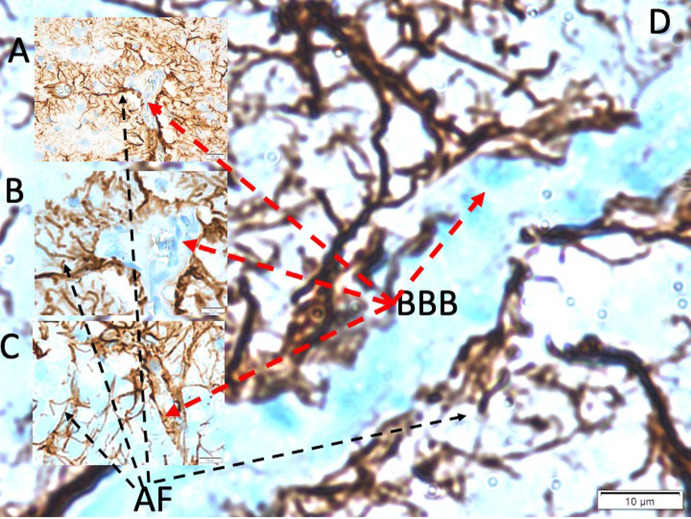
A normal blood–brain barrier formed by an artery with normal vascularity surrounded by dense astrocytes in the hippocampus is seen in the control group. In the sham group, a slightly narrowed artery and the blood–brain barrier with minimally fragmented astrocyte footpads are observed. In the study group, a large number of fragmented astrocytes and a blood–brain barrier with impaired wall integrity are observed.

Normal cell density was significantly decreased despite a significant increase in apoptotic cell density in the CPX group after SAH. Flat epithelial cells, angulations, elongated irregular nuclei, cellular shrinkage, and cytoplasmic condensation were observed in group 3 (Figures 1-5).

In the histopathological examinations of the cerebrums, thromboembolic occlusion was detected in only 1 artery of test subjects in the sham group. In branches of the middle cerebral artery, occlusion exists in 2 test subjects that did not survive in group 3 and in 1 of the surviving animals. The mean number of arteries occluded with thromboembolic particles was higher in group 3 than in the sham and control groups. There was a significant difference between the number of occluded arteries in the surviving and non-surviving animals (*P* < .05). However, the difference between groups 2 and 3 was not significant (*P* > .05).

### Numerical Findings

The number of degenerated epithelial cells in the CPX and the number of thromboembolisms in the hippocampal arteries were 7 ± 2 and 1 ± 1 in group 1, 16 ± 4 and 3 ± 1 in group 2, and 64 ± 9 and 6 ± 2 in group 3, respectively. The significance levels were *P* < .005 for group 1 vs. group 2, *P* < .0005 for group 2 vs. group 3, and *P* < .00001 for group 1 vs. group 3.

## Discussion

To maintain normal cerebral blood flow, the morphology of the subarachnoid space through which the cerebral arteries travel and the volume and content of the CSF must be within the normal ranges. Choroidal artery vasospasm after SAH causes decreased blood flow and damage to CPX.^1^ For cerebral arteries to fulfill their dynamic, kinetic, and rheological functions, they should be able to pulse easily within the subarachnoid space.^2^ Cerebrospinal fluid is also of great importance for the thermodynamic functions of arteries and brain tissues and is critical in the regulation of osmotic and oncotic pressure in the arteries and the normalization of hematocrit values. In cases of SAH, disruption of the mechanical and thermodynamic balance of the subarachnoid space leads to disruption of vascular dynamism and rheological functions. After SAH, innervation of the CPX has disrupted and CSF secretion decreases as a result of damage to the trigeminal, facial, glossopharyngeal, and vagal nerves.^2^ Vascular hemodynamics deteriorate secondary to CPX degeneration, thromboembolic events increase at the arteriolar level due to decreased CSF in the perivascular area, and an increase in intracranial pressure occurs.^4^ Consequently, increased cerebral edema that develops due to thromboembolism, which cannot be normalized, can lead to brain death. Cerebral venous thrombosis is an important complication of spontaneous CSF leakage.^5^ Cerebral venous thrombosis following CSF leakage after spinal anesthesia is a rare but serious complication, because CSF drainage may alleviate venous sinus occlusion by regulating sinus blood flow.^6,7^ Cisternal irrigation may be beneficial in cases of meningitis because of its protective effects on the renormalization of cerebral circulation.^8^

Choroid plexus is an intraventricular structure composed of single-row ciliated cuboid epithelial cells covered with numerous villi that secrete CSF. Collagen fibers, sparse dendritic cells, macrophages, fibroblasts, and large capillaries have windowed endothelium.^1^ These structures secrete significant amounts of CSF, synthesize a large number of molecules, transport nutrients from the blood to the CSF, and recycle by-products of brain metabolism. They absorb materials as necessary and participate in the immune surveillance of the brain. Choroid plexus is richly innervated by cholinergic, adrenergic, peptidergic, and serotoninergic fibers.^2^ Cerebrospinal fluid secretion decreases by more than half with aging, especially in Alzheimer’s disease. The blood–CSF barrier in CPX significantly protects neuronal and vascular structures.^3^ As an immune sensor for the brain, CPX performs immunological and signal transduction functions in the central nervous system via CSF. Choroid plexus is also an active site for protein synthesis and contains several receptors involved in inflammatory processes, including bacterial lipopolysaccharides, neurotransmitters, growth factors, and hormones. Choroidal epithelial cells transport glucose, anti-inflammatory corticosteroids, and phospholipids from blood to the CSF.^4,5^ Choroid tissue constitutes the hematencephalic barrier. The metabolic activity of CPX is estimated to be half of that of the kidneys.^6^ Plexuses secrete approximately 90% of the CSF, and the remaining 10% originates from the brain interstitial fluid drainage. Choroid epithelial cells synthesize numerous proteins, including transferrin, ceruloplasmin, cytokines, and growth factors.^7,15^ Choroid plexus actively transports numerous molecules from the blood to the CSF, including folate, glucose, and vitamins B6, B12, and C, and possibly E.^8^

The composition of the CSF differs from that of the plasma; the pH of the CSF is slightly acidic, similar to that of the interstitial fluid of the brain. The levels of Na^+^, K^+^, Ca^++^, HCO^3–^, proteins, and glucose are lower, and Cl^–^ and Mg^++^ are higher in the CSF than in the plasma.^1^ Folate levels are 2-3 times higher in the CSF than in plasma.^2^

Psammoma bodies occur with atrophy of epithelial cells and are particularly observed in aging individuals.^4^ The mean CSF production rate is significantly decreased in Alzheimer’s and Parkinson’s diseases.^5^ Choroid plexus acts as a CSF–blood barrier. Ischemic CPX can cause meningocerebral inflammation, whereas SAH causes choroidal artery vasospasm and ischemic degeneration of CPX.^6^ Cerebrospinal fluid secretion may increase in the early stages of SAH, and disruption of glossopharyngeal discharges in the late stages of SAH may decrease CSF release.^7^

Choroid plexus cysts are the most common cause of hydrocephalus in the elderly but not in children because they do not contain water vesicles.^8,16^ Acidosis of the blood and CSF causes CPX degeneration.^9^ If SAH extends to the brain ventricles, ischemic degeneration occurs in the CPX.^6^ Morphological changes, such as shrinkage, condensation, and angulation of CPX cells, are recognized as the criteria for CPX degeneration.^10^ Acute hydrocephalus occurs within 72 hours as a common complication of SAH. Irrigation can be used to prevent thrombus formation and to clear the subarachnoid space to prevent thrombus formation.^11^ Although various etiological theories blame ependymal desquamation, acidosis-related CPX damage is a newly described factor in cerebral thrombus formation.^12^

### Limitation

This study does not contains angiographic data because this is only a histopathological study.

## Conclusion

Irrigation procedures to prevent thrombus development can be used to reduce the incidence and severity of SAH-related complications by removing the collected inflammatory materials from the subarachnoid spaces and vessels. This study demonstrated that decreased CSF levels due to CPX degeneration caused previously unrecognized cerebral thromboembolism following SAH. Cerebrospinal fluid exchange or cerebrospinal dialysis could be used as new treatment modality in the future.

## References

[1] Yilmaz A Aydin MD Kanat A et al. The effect of choroidal artery vasospasm on choroid plexus injury in subarachnoid hemorrhage: an experimental study. Turk Neurosurg. 2011;21(4):477 482. (10.5137/1019-5149.JTN.4204-11.1) 22194103

[2] Aydin MD Kanat A Turkmenoglu ON Yolas C Gundogdu C Aydın N . Changes in number of water-filled vesicles of choroid plexus in early and late phase of experimental rabbit subarachnoid hemorrhage model: the role of petrous ganglion of glossopharyngeal nerve. Acta Neurochir (Wien). 2014;156(7):1311 1317. (10.1007/s00701-014-2088-7) 24752726

[3] Aydin MD Kotan D Aydin N Gundogdu C Onder A Akcay F . Mechanism of cerebral fat embolism in subarachnoid hemorrhage: an experimental study. Neuropathology. 2006;26(6):544 549. (10.1111/j.1440-1789.2006.00733.x) 17203591

[4] Kotan D Aydin MD Gundogdu C Aygul R Aydin N Ulvi H . Parallel development of choroid plexus degeneration and meningeal inflammation in subarachnoid hemorrhage - experimental study. Adv Clin Exp Med. 2014;23(5):699 704. (10.17219/acem/37221) 25491682

[5] Bisinotto FMB Dezena RA Abud TMV Martins LB . Cerebral venous thrombosis after spinal anesthesia: case report. Rev Bras Anestesiol. 2017;67(3):305 310. (10.1016/j.bjan.2014.09.005) 25840468

[6] Bujoreanu I Ferguson M Saleh H . Chemotherapy associated dural sinus thrombosis presenting as a cerebrospinal fluid leak. BMJ Case Rep. 2020;13(6). (10.1136/bcr-2020-235240) PMC7265007 32487533

[7] Chang T Yang YL Gao L Li LH . Cerebral venous sinus thrombosis following transsphenoidal surgery for craniopharyngioma: a case report. World J Clin Cases. 2020;8(6):1158 1163. (10.12998/wjcc.v8.i6.1158) 32258087 PMC7103970

[8] Bienfait HP Gijtenbeek JM van den Bent MJ de Bruin HG Voogt PJ Pillay M . Cerebral venous and sinus thrombosis with cerebrospinal fluid circulation block after the first methotrexate administration by lumbar puncture. Neuroradiology. 2002;44(11):929 932. (10.1007/s00234-002-0854-3) 12428129

[9] Kalita J Singh RK Misra UK Kumar S . Evaluation of cerebral arterial and venous system in tuberculous meningitis. J Neuroradiol. 2018;45(2):130 135. (10.1016/j.neurad.2017.09.005) 28970078

[10] Hoelscher C Sweid A Ghosh R et al. Cerebral deep venous thrombosis and COVID-19: case report. J Neurosurg. 2020:1 4. (10.3171/2020.5.JNS201542) 32886922

[11] Utku U Gokce M Senoglu M . Cerebral venous sinus thrombosis associated with spontaneous intermittent cerebrospinal fluid rhinorrhea: a case report. Med Princ Pract. 2012;21(4):392 394. (10.1159/000336782) 22487926

[12] Park JH Yoon SH . New concept of cerebrospinal fluid dynamics in cerebral venous sinus thrombosis. Med Hypo. 2008;70(1):143 147. (10.1016/j.mehy.2007.03.036) 17570605

[13] Aydin MD Gündogdu C Akçay F Gursan N . Protective effects of cisternal irrigation on leptomeningeal and cortical structures in meningitis: an experimental study. Neurol India. 2005;53(1):90 92. (10.4103/0028-3886.15069) 15805663

[14] Fang Y Huang L Wang X et al. A new perspective on cerebrospinal fluid dynamics after subarachnoid hemorrhage: from normal physiology to pathophysiological changes. J Cereb Blood Flow Metab. 2022;42(4):543 558. (10.1177/0271678X211045748) 34806932 PMC9051143

[15] Liszczak TM Black PM Tzouras A Foley L Zervas NT . Morphological changes of the basilar artery, ventricles, and choroid plexus after experimental SAH. J Neurosurg. 1984;61(3):486 493. (10.3171/jns.1984.61.3.0486) 6747684

[16] Lolansen SD Rostgaard N Barbuskaite D et al. Posthemorrhagic hydrocephalus associates with elevated inflammation and CSF hypersecretion via activation of choroidal transporters. Fluids Barriers CNS. 2022;19(1):62. (10.1186/s12987-022-00360-w) PMC9367104 35948938

